# Thoughts about health and patient-reported outcomes among people with diabetes mellitus: results from the DiaDec-study

**DOI:** 10.1186/s12889-021-10231-y

**Published:** 2021-01-26

**Authors:** Sandra O. Borgmann, Nadja Chernyak, Burkhard Haastert, Ute Linnenkamp, Silke Andrich, Rabea Schlenker, Oliver Razum, Andrea Icks

**Affiliations:** 1 Institute for Health Services Research and Health Economics, German Diabetes Center (DDZ), Leibniz Center for Diabetes Research at the Heinrich Heine University Düsseldorf, Auf’m Hennekamp 65, 40225 Düsseldorf, Germany; 2 Institute for Health Services Research and Health Economics, Centre for Health and Society, Faculty of Medicine, Heinrich Heine University Düsseldorf, Düsseldorf, Germany; 3 German Center for Diabetes Research (DZD), Munich-Neuherberg, Germany; 4 mediStatistica, Neuenrade, Germany; 5 School of Public Health, AG 3 Epidemiologie & International Public Health, Bielefeld University, Bielefeld, Germany

**Keywords:** Patient-centered care, Diabetes mellitus, Patient reported outcomes, Health-related quality of life, Thinking

## Abstract

**Background:**

There is considerable evidence that repetitive negative thoughts are often associated with adverse health outcomes. The study aims are (i) to identify the frequency and valence of thoughts about health in people with diabetes mellitus using questions based on the day reconstruction method (DRM) and (ii) to analyse associations between thoughts about health and health-related quality of life (HRQoL), diabetes-related distress and depressive symptoms.

**Methods:**

Cross-sectional study of a random sample of a German statutory health insurance population with diabetes aged between 18 and 80 linking questionnaire and claims data. Associations between frequency and valence of thoughts about health on a previous day and HRQoL assessed by a 12-Item Short-Form Health Survey, diabetes-related distress assessed using the Problem Areas in Diabetes scale and depressive symptoms assessed by Patient Health Questionnaire-9 were analysed using linear and logistic regression analysis, adjusting for sociodemographic and clinical characteristics.

**Results:**

Thoughts about health were analysed in 726 participants (86% type 2 diabetes, 62% male, mean age 67.6 ± 9.7 years). A total of 46% had not thought about their health the day before, 17.1% reported low frequency and negative thoughts, 21.4% low frequency and positive thoughts, 12.1% high frequency and negative thoughts and 3.4% high frequency and positive thoughts. The presence of thoughts about health irrespective of their frequency and valence is associated with a lower physical and mental component summary score of the 12-Item Short-Form Health Survey. Negative thoughts are associated with high diabetes-related distress. Frequent or negative thoughts are associated with depressive symptoms.

**Conclusions:**

Thoughts about health are a part of everyday life for a substantial number of people with diabetes. Surprisingly, even positive thoughts are associated with poorer HRQoL in our study. Further research within the DRM paradigm is needed to understand how thoughts about health may affect people’s (assessment of) state of health. Thoughts about health should be considered in diabetes education and patient counselling with a view to preventing and treating emotional disorders. More attention should be paid to the outcomes of interventions that may themselves lead to an increase in the frequency of thoughts about health.

**Supplementary Information:**

The online version contains supplementary material available at 10.1186/s12889-021-10231-y.

## Background

Diabetes mellitus (DM) is a chronic condition and is associated with complex and time-consuming self-management tasks in everyday life, increased morbidity, and reduced health-related quality of life (HRQoL) [1].

A systematic review found evidence that perseverative negative thinking (e.g., worries and rumination) was associated with depression, anxiety and emotional distress in people with long-term conditions [2]. Moreover, evidence suggests that repetitive negative thinking (RNT) plays a causal role in the development and/or persistence of emotional disorders [3]. Thoughts about health in people with DM can arise not only from increased mortality and comorbidity risks, but also from self-management and its consequences [4, 5]. Negative thoughts (referred to as worries) about weight amongst people with DM are significantly associated with higher perceived diabetes-related distress and poorer psychological well-being [6]. Another study showed that a higher frequency of negative thoughts was associated with higher levels of depressive symptoms in people with DM [7]. The authors used the validated Automatic Thoughts Questionnaire which measured depression-related cognitions by assessing negative self-statements (e.g., ‘I feel like I’m up against the world’) on a 5-Likert scale from ‘not at all’ to ‘all the time’ [7–9].

Repetitive thinking may be adaptive (e.g. planning), benign (e.g. day dreaming) or maladaptive (worry or rumination) [10]. The prevailing valence of thoughts (negative or positive) often determines whether repetitive thoughts are helpful or unhelpful [3]. Perseverative cognition, defined as rumination and worries, may increase the risk of developing long-term somatic and psychological disease outcomes [2, 11, 12]. In contrast, positive thoughts play an important role in achieving well-being and in the reduction of negative effects of stressors [13–15]. Although the valence of the thoughts typically matches the associated affect [3], some studies found that negative thoughts (e.g. defensive pessimism) and positive thoughts (e.g. unrealistic optimism) can have the opposite effect on health outcomes [3, 15]. Other authors indicate that the concurrent experience of mixed emotions (negative and positive) seems to be essential in improving long-term health outcomes such as psychological well-being [16, 17].

Despite an increasing interest in the process of RNT in clinical literature, research with a truly ‘transdiagnostic’ or generic perspective on this topic - as opposed to the disease-specific approach focussing on repetitive thinking such as anxious worry or depressive rumination in isolation - is still scarce [10]. Some scales have been developed to assess RNT across disorders, for example the Perseverative Thinking Questionnaire [18] and the Repetitive Thinking Questionnaire [19]. These self-report questionnaires ask respondents about the typical content and/or style of their thinking referring to a retrospective period and usually assess general tendencies or traits. Such questionnaires have recently been criticised for reflecting a constructed experience that is biased by time, (metacognitive) beliefs, and state factors, rather than reflecting the actual experience or behaviour [20]. Another shortcoming of these instruments is their focus on negative thoughts. It is conceivable that people who are asked to think about negative aspects are more aware of these aspects at the time of the survey than in everyday situations [21].

There is increasing interest in measuring experiences (e.g. feelings and thoughts) and contextual information using the Ecological Momentary Assessment (EMA) [22] and the Day Reconstruction Method (DRM) [23]. EMA is based on reports at specific (often randomly chosen) points in time. The DRM, which is used to approximate the more expensive EMA, asks people to write a diary of the main episodes of the previous day and recall the type and intensity of feelings experienced during each activity [23]. A benefit of the DRM seems to be accurate emotional recall when respondents are asked to retrieve specific recent situations, thus reducing recall bias [23, 24].

Dolan (2011) used questions based on DRM to assess frequency and valence of thoughts about health. In a cross-sectional study, he identified an association between preference-based HRQoL and thoughts about health [21]. Participants with frequent and positive thoughts rated their state of health better, adjusting for their current state of health state. Those with high frequency and negative thoughts about health had significantly lower utility values, i.e. were willing to sacrifice more years of life to improve their current state of health.

The present study used the DRM-based questions on thoughts about health from the study conducted by Dolan (2011) to collect data on frequency and valence of general, non-specific thoughts about health close to everyday life and to analyse associations between thoughts about health and HRQoL, diabetes-related distress and depressive symptoms. It was therefore possible to examine the association of positive thoughts and patient-reported health outcomes, which is still poorly investigated in people with DM.

## Methods

### Study design and population

The present cross-sectional study included data from participants who took part in the ‘Quality of life, disability, health care utilisation and costs in patients with diabetes: The role of depression’ study (DiaDec-study) [25]. The DiaDec-study sought to estimate the prevalence of diagnosed and undiagnosed depression in people with DM, to compare costs and health care utilisation, and to analyse HRQoL in people with DM with and without co-morbid depression. The participants were a random sample of the ‘pronova Betriebskrankenkasse’ (pronova BKK), a German statutory health insurance, aged between 18 years and 80 years and with a diabetes diagnosis (response 51%). Diabetes diagnosis was based on the ‘International Statistical Classification of Diseases and Related Health Problems 10th Revision (ICD-10)’, antihyperglycaemic medication and HbA_1c_ values, as described as diagnostic criteria in other published studies [26, 27]. Inclusion criteria were described in detail in the study protocol [25]. Data assessment comprises questionnaire data and individually linked longitudinal statutory health insurance data, 1 year before and 1 year after the survey.

The present study applied a similar approach to Dolan (as described below) to analyse data elicited by questions on thoughts about health. The questions were incorporated into the questionnaire at a later stage of the study, which was sent out once to the participants. Thus, of the 1860 participants who sent back their questionnaire 783 participants sent back a questionnaire including questions on ‘thoughts about health’. Fifty-seven participants were excluded due to missing data in the ‘thoughts about health’ instrument, resulting in a study population of 726 participants.

### Assessment of thoughts about health

The questions on thoughts about health (Additional file 1, Appendix 1) are based on the DRM and were extracted from the study by Dolan [21] (2011) and translated into German.

The first question enquires about how often participants thought about their health the day before (reference to a single day). Participants can answer by specifying the frequency (‘not at all’, ‘a few times’, ‘many times’ and ‘continually’). The second question refers to the intensity of certain feelings that can occur during the presence of thoughts: ‘If you thought about your health yesterday, how did you feel about those thoughts?’. On a scale of 0 (does not apply at all) to 6 (applies exactly), answers can be given to a total of three negative feelings (angry, depressed and worried) and one positive feeling (happy). Three frequency groups were defined. The first group included all participants who had not thought about their health the day before. Respondents who thought about health ‘a few times’ were placed in the low frequency group, and those who thought about health ‘many times’ or ‘continually’ were placed in the high frequency group. As in the Dolan study [21] the ‘U-index method’ reported by Kahneman et al. (2004) [23] was used to determine the valence of thoughts. If ‘happy’ was the highest or joint-highest rated feeling, thoughts were labelled as ‘positive’; otherwise thoughts were labelled as ‘negative’.

Five groups were defined by combining these two group allocations: ‘no thoughts about health’, ‘low frequency and positive thoughts’, ‘high frequency and positive thoughts’, ‘low frequency and negative thoughts’ and ‘high frequency and negative thoughts’.

### Outcome assessment

The 12-Item Short-Form Survey (SF-12) measures HRQoL and is a short version of the SF-36 [28]. The instrument was tested in people with DM and is available and validated in German [29, 30]. The SF-12 contains 12 questions representing eight health dimensions, e.g. physical function and emotional role function. The dimensions reflect a physical and mental component summary score of the SF-12.

The Problem Areas in Diabetes (PAID) scale identifies diabetes-related distress in people with DM [31, 32]. The instrument was validated in German [33] and is used to measure diabetes-specific HRQoL and also as a screening instrument for depressive symptoms among people with DM [31]. This instrument consists of 20 items relating to diabetes-specific stress situations. Each item can be answered on a 5-Likert scale from ‘no problem’ [0] to ‘major problem’ [4]. Diabetes-related distress was coded as ‘high diabetes-related distress’ (cut-off score ≥ 40) as recommended [31].

Depressive symptoms were assessed using the Depression Module of the Patient Health Questionnaire (PHQ-9) [34]. The PHQ-9 has been validated in studies with people with DM and in German [34–37]. Participants answer how often they have felt affected by nine different conditions over the previous 2 weeks. The following four answers can be given: ‘not at all’ [0], ‘several days’ [1], ‘more than half the days’ [2] and ‘nearly every day’ [3] [34]. We used the categorical diagnostic algorithm to obtain an indication of clinically relevant depression [38]. This adds a clinical perspective to the results of the other patient-reported outcomes. ‘Depressive symptoms’ were coded if at least two questions were answered with ‘more than half the days’ or ‘nearly every day’ and if item one (‘little interest or pleasure in doing things’) or item two (‘feeling down, depressed or hopeless’) were included. Item nine (‘thoughts that you would be better off dead or of hurting yourself in some way’) was also counted if ‘several days’ were reported [34, 37, 39].

### Other variables

The following variables, which previous studies have found to be associated with repetitive thoughts (e.g. worries and rumination) and specific thoughts at the time of the diabetes diagnosis (e.g. ‘nothing I could do about complications’), were included in our study: age, sex, education, type of diabetes, diabetes duration and comorbidity [6, 13, 40–42]. Most of these variables were collected in the DiaDec-study survey in 2013. Education was coded as ‘no graduation’, ‘other graduation’ and ‘university degree’, type of diabetes was coded as ‘type 1’, ‘type 2’, ‘other’ and ‘not known to participants’, and diabetes duration was coded as ‘less than 10 years’ and ‘10 or more years’. Comorbidity was measured using the health insurance data in 2012 based on the results of the 80 hierarchical morbidity groups according to the German morbidity-oriented risk structure compensation scheme (total number of comorbidities) [43].

Further variables which might be associated with thoughts about health were also included, i.e. characteristics related to socioeconomic status such as employment and marital status. In addition to comorbidities, people’s own perception of their state of health is also relevant. This variable was therefore added. Furthermore, the mode of diabetes treatment was included, as it may indicate the severity of the disease. Various requirements, for instance remembering to take medication, can also lead to treatment being perceived more intensely in everyday life.

The 2013 survey coded employment as ‘yes’ or ‘no’. Marital status was coded as ‘single’, which also included people who are widowed, divorced or live permanently separated, and by ‘married and living together’. Migration background was coded as ‘yes’ if the participant or at least one parent did not possess German nationality by birth, otherwise it was coded as ‘no’ [44]. The ‘In general, would you say your health is … ’ item of the SF-12 questionnaire, coded as ‘excellent’, ‘very good’, ‘good’, ‘fair’ and ‘poor’, was selected to assess perceived health status. Mode of diabetes treatment was identified using available drug information in the 2012 health insurance data and coded in relation to oral glucose-lowering drugs and insulin as ‘yes’ or ‘no’.

### Data analysis

Descriptive summaries for patient characteristics were obtained (depending on the distribution of the variables by frequencies, percentages, means (M) ± standard deviations (SD)). We compared participant characteristics between the ‘thought groups’.

Multivariate linear and logistic regression models were fitted to assess associations between thought groups and outcomes. These models estimate regression coefficients (betas) and odds ratios (OR) with 95% confidence intervals (CI). Separate models were fitted for the outcomes, using the physical and mental summary score of the SF-12 as metric dependent variables and diabetes-related distress (low vs. high) and depressive symptoms (yes vs. no) as dependent binary variables. In addition, we checked for potential artefacts from dichotomising the variables ‘diabetes-related distress’ and ‘depressive symptoms’ and performed sensitivity analyses with the respective score. Two final models were considered for each outcome, one only including the four thought groups as independent variables vs. no thoughts, the other including all preselected sociodemographic and health-related variables: age, sex, education (other graduation or university degree vs. no graduation), employment (yes vs. no) and marital status (married and living together vs. others) and the following health-related variables: diabetes duration (≥10 years), mode of diabetes treatment (oral glucose-lowering drugs (yes vs. no), insulin (yes vs. no)) and comorbidity (> 3 comorbidities). Perceived health status (fair to poor vs. good to excellent) was added for the PAID and PHQ-9 outcomes. Type of diabetes was excluded because of a high number of unknown type or missings.

Data analysis was performed using SAS software, Version 9.4 (SAS Institute Inc., Cary, NC). The significance level was 5% and two-sided if not stated otherwise.

## Results

### Participant characteristics

The present study population (*n* = 726) included more male participants (Table 1). The majority of participants (94.7%) had no university degree. Three of four were currently not working and were married. One in six people had a migration background. Most participants (85.6%) stated that they had type 2 diabetes. Half of the participants were diagnosed ten or more years ago. Two thirds were treated with oral glucose-lowering drugs and about one third with insulin. The number of comorbidities varied from zero to 17 and most participants considered their general state of health to be good.

**Table 1:** Participants’ characteristics

Characteristics	n (%) / M ± SD; median (quartile)
Total number of participants	726
Age (years), *n* = 726	67.6 ± 9.7; 70.0 (q1: 62.0; q3: 75.0)
Sex, *n* = 726	
Female	273 (37.6)
Education, *n* = 707	
No graduation	146 (20.7)
Other graduation	523 (74.0)
University degree	38 (5.4)
Employment, *n* = 711	177 (24.9)
Marital status, *n* = 722	
Married and Living together	543 (75.2)
Migration background, *n* = 724	120 (16.6)
Type of diabetes, *n* = 720	
Type 1 diabetes	59 (8.2)
Type 2 diabetes	616 (85.6)
Other	7 (1.0)
Not known to participants	38 (5.3)
Diabetes duration, *n* = 702	
10 years or more	349 (49.7)
Oral glucose-lowering drugs, *n* = 726	496 (68.3)
Insulin, *n* = 726	213 (29.3)
No. of comorbidities, *n* = 726	
> 3 comorbidities	311 (42.8)
No. of comorbidities	3.7 ± 2.1; 3.0 (q1: 2.0; q3: 5.0)
Perceived health status, *n* = 722	
Very good–excellent	62 (8.6)
Good	406 (56.2)
Fair–poor	254 (35.2)
Physical summary score of theSF-12, *n* = 660	41.9 ± 11.0; 43.7 (q1: 33.1; q3: 51.7)
Mental summary score of theSF-12, *n* = 660	50.1 ± 10.5; 53.7 (q1: 42.3; q3: 58.1)
High diabetes-related distress,*n* = 678	92 (13.6)
Depressive symptoms, *n* = 722	85 (11.8)

### ‘Thoughts about health’ groups

Group classification was carried out for 726 participants. This included those 334 participants (46.0%) who had not thought about their health the day before. A total 124 participants (17.1%) were assigned to the ‘low frequency and negative thoughts’ group and 155 participants (21.4%) to the ‘low frequency and positive thoughts’ group. The ‘high frequency and negative thoughts’ group included 88 participants (12.1%) and the ‘high frequency and positive thoughts’ group included 25 participants (3.4%). The characteristics of the participants within the thought groups are shown in Table 2.

**Table 2:** Participants’ characteristics in the thought groups

Characteristics	‘no thoughts’ group (*n* = 334)	‘low frequency and negative thoughts’ group (*n* = 124)	‘low frequency and positive thoughts’ group (*n* = 155)	‘high frequency and negative thoughts’ group (*n* = 88)	‘high frequency and positive thoughts’ group (*n* = 25)
	n (%) / (M ± SD)	n (%) / (M ± SD)	n (%) / (M ± SD)	n (%) / (M ± SD)	n (%) / (M ± SD)
Age (years)	68.4 ± 8.9	67.4 ± 10.1	68.6 ± 9.7	63.4 ± 11.0	65.5 ± 9.4
Female	127 (38.0)	52 (41.9)	53 (34.2)	28 (31.8)	13 (52.0)
Education					
*No graduation*	61 (18.8)	33 (26.8)	21 (13.9)	25 (29.4)	6 (25.0)
*Other graduation*	248 (76.5)	81 (65.9)	123 (81.5)	54 (63.5)	17 (70.8)
*University degree*	15 (4.6)	9 (7.3)	7 (4.6)	6 (7.1)	1 (4.2)
Employment	77 (23.7)	28 (22.8)	32 (21.2)	31 (35.6)	9 (36.0)
Married and living together	260 (78.3)	93 (75.6)	114 (74.0)	60 (68.2)	16 (64.0)
Migration background	43 (12.9)	18 (14.6)	29 (18.7)	25 (28.4)	5 (20.0)
Type of diabetes					
*Type 1 diabetes*	21 (6.4)	12 (9.7)	13 (8.4)	11 (12.6)	2 (8.0)
*Type 2 diabetes*	294 (89.1)	95 (76.6)	135 (87.7)	70 (80.5)	22 (88.0)
*Other*	2 (0.6)	3 (2.4)	0 (0.0)	2 (2.3)	0 (0.0)
*Not known to participants*	13 (3.9)	14 (11.3)	6 (3.9)	4 (4.6)	1 (4.0)
Diabetes duration (10 ≤ years)	150 (46.7)	64 (53.3)	80 (52.6)	42 (50.0)	13 (52.0)
Oral glucose-lowering drugs	237 (71.0)	86 (69.4)	102 (65.8)	54 (61.4)	17 (68.0)
Insulin	79 (23.7)	37 (29.8)	48 (31.0)	37 (42.0)	12 (48.0)
No. of comorbidities	3.3 ± 1.6	3.8 ± 2.0	3.9 ± 2.3	4.4 ± 2.5	4.5 ± 3.3
Perceived health status					
*Very good–excellent*	44 (13.1)	4 (3.2)	13 (8.3)	0 (0.0)	1 (4.0)
*Good*	240 (71.9)	48 (39.0)	95 (61.3)	13 (15.3)	10 (40.0)
*Fair–poor*	50 (15.0)	71 (57.8)	47 (30.3)	72 (84.7)	14 (56.0)
Physical summary score of the SF-12	46.5 ± 9.1	36.9 ± 9.5	42.3 ± 10.8	31.8 ± 8.8	33.8 ± 12.1
Mental summary score of the SF-12	54.5 ± 7.6	44.9 ± 10.5	52.4 ± 8.5	37.6 ± 9.9	43.4 ± 11.3
High diabetes-related distress	12 (3.9)	28 (23.7)	11 (7.5)	36 (46.2)	5 (20.0)
Depressive symptoms	11 (3.3)	23 (18.5)	3 (1.9)	40 (45.5)	8 (33.3)

### Patient-reported outcomes

**Fig. 1:**
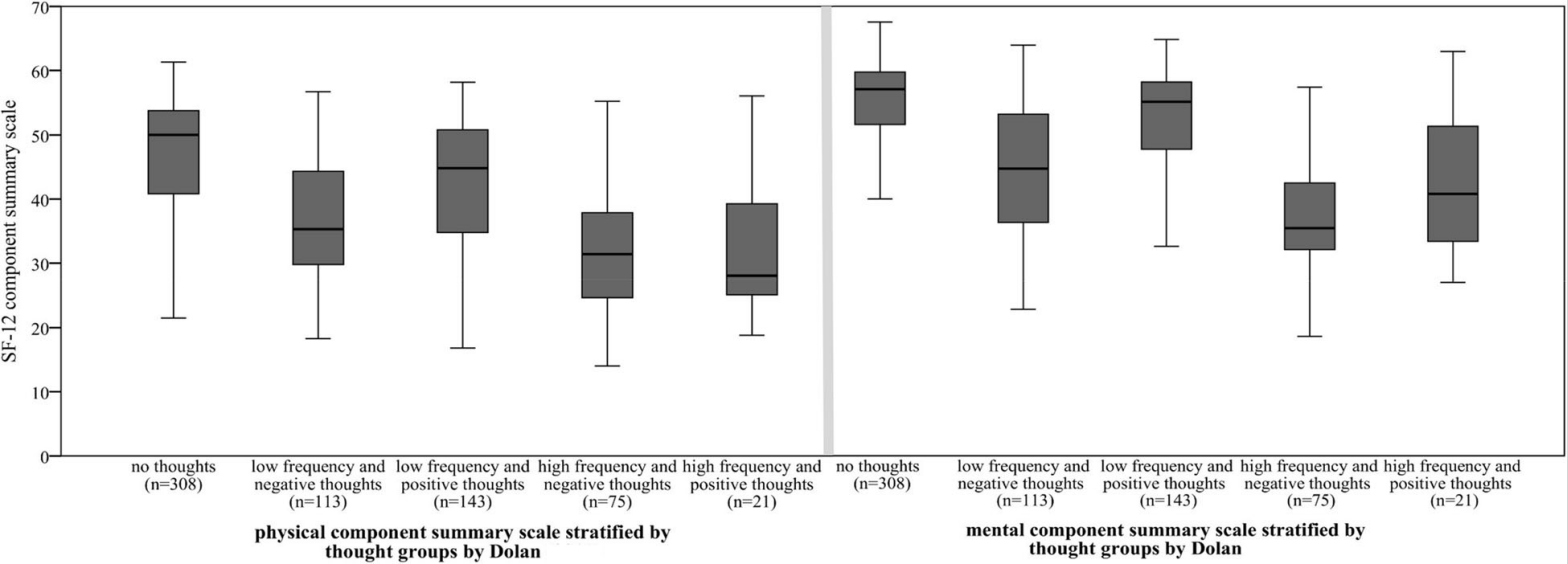
Box plot comparing SF-12 physical and mental summary scale in participants stratified by thought groups (*n* = 660). Boxplots show the median (horizontal line), interquartile range (box), and range (whiskers)

**Fig. 2:**
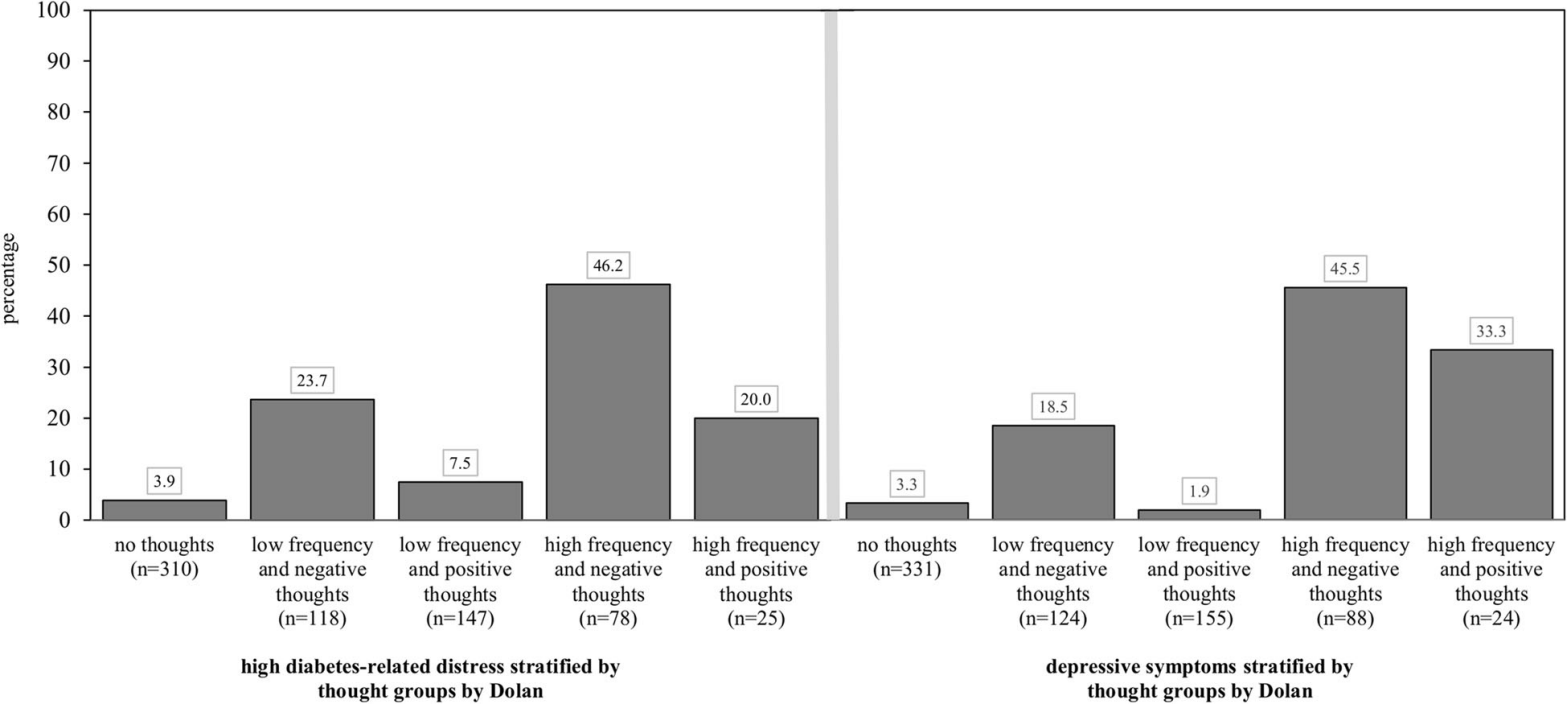
Percentages of high diabetes-related distress (PAID) and depressive symptoms (PHQ-9) in participants stratified by thought groups

Table 1 shows the mean and standard deviation of both summary scores of the SF-12 in the study population. The physical summary score and the mental summary score (Fig. 1) of the SF-12 differed between the thought groups. Participants in the ‘no thoughts’ group showed the highest mean scores in both scales compared to the other four groups. In contrast, the ‘high frequency and negative thoughts’ and ‘high frequency and positive thoughts’ groups showed the lowest mean scores in both scales. High diabetes-related distress was identified in 13.6% and depressive symptoms were present in 11.8% of the study population. As illustrated by Fig. 2, depressive symptoms and high diabetes-related distress were most common in people with high frequency and negative thoughts. Depressive symptoms and high diabetes-related distress were more common in the ‘low frequency and negative thoughts’ and ‘high frequency and positive thoughts’ groups than in the ‘low frequency and positive thoughts’ and ‘no thoughts’ groups.

### Regression analysis

Univariate linear regression models indicate that participants who reported to have thoughts about health were significantly more likely to have a lower physical and mental component summary score of the SF-12 compared to the reference group of participants with no thoughts (Table 3). Adjustment for sociodemographic and health-related variables did not alter ORs substantially. The univariate logistic regression model suggests that negative thoughts as well as high frequency and positive thoughts were significantly associated with high diabetes-related distress (Table 4). Multiple adjusted negative thoughts with high or low frequency remained significantly associated with diabetes-related distress. Participants who reported highly frequent negative or positive thoughts as well as low frequency and negative thoughts were significantly more likely to present depressive symptoms. Similar results were found after adjustment. In the two sensitivity analyses, we saw that linear regression models for diabetes-related distress and depressive symptoms lead to associations comparable to those presented in Table 4. Within the sensitivity analyses we saw a trend to stronger associations. This indicates, that our results are stable.

**Table 3:** Linear regression models, HRQoL

	Physical summary score of the SF-12			Mental summary score of the SF-12		
	Beta	95%-CI	*p*-value	Beta	95%-CI	*p*-value
**Univariate model**	*n* = 660			*n* = 660		
No thoughts	0.00			0.00		
Low frequency and negative thoughts	−9.56	[−11.64; −7.47]	**< 0.001**	−9.52	[−11.42; −7.63]	**< 0.001**
Low frequency and positive thoughts	−4.22	[−6.14; −2.30]	**< 0.001**	−2.10	[−3.84; −0.36]	**0.018**
High frequency and negative thoughts	−14.65	[−17.09; −12.21]	**< 0.001**	−16.88	[−19.10; −14.67]	**< 0.001**
High frequency and positive thoughts	−12.67	[−16.95; −8.40]	**< 0.001**	−11.11	[−14.99; −7.23]	**< 0.001**
**Multivariate model**^**a**^	*n* = 624			*n* = 624		
No thoughts	0.00			0.00		
Low frequency and negative thoughts	−8.96	[−11.00; −6.93]	**< 0.001**	−9.20	[−11.15; −7.25]	**< 0.001**
Low frequency and positive thoughts	−3.56	[−5.43; −1.70]	**< 0.001**	−2.02	[−3.82; −0.23]	**0.027**
High frequency and negative thoughts	−14.25	[−16.75; −11.75]	**< 0.001**	−15.57	[−17.98; −13.17]	**< 0.001**
High frequency and positive thoughts	−11.32	[−15.51; −7.13]	**< 0.001**	−9.91	[−13.95; −5.89]	**< 0.001**

*SF-12* 12-Item Short-Form Survey, *CI* confidence interval

^a^adjusted for sociodemographic (age, sex, education, employment, marital status) and health-related variables (diabetes duration ≥10 years, oral glucose-lowering drugs, insulin, number of comorbidities > 3)

**Table 4:** Logistic regression models, diabetes-related distress and depressive symptoms

	Diabetes-related distress			Depressive symptoms		
	OR	95%-CI	*p*-value	OR	95%-CI	*p*-value
**Univariate model**	*n* = 678			*n* = 722		
No thoughts	1.00			1.00		
Low frequency and negative thoughts	7.73	[3.78; 15.81]	**< 0.001**	6.62	[3.12; 14.06]	**< 0.001**
Low frequency and positive thoughts	2.01	[0.86; 4.67]	0.105	0.57	[0.16; 2.09]	0.400
High frequency and negative thoughts	21.29	[10.27; 44.12]	**< 0.001**	24.24	[11.65; 50.46]	**< 0.001**
High frequency and positive thoughts	6.21	[1.99; 19.36]	**0.002**	14.55	[5.14; 41.15]	**< 0.001**
**Multivariate model**^**a**^	*n* = 636			*n* = 672		
No thoughts	1.00			1.00		
Low frequency and negative thoughts	4.57	[2.08; 10.05]	**< 0.001**	3.16	[1.39; 7.17]	**0.006**
Low frequency and positive thoughts	1.40	[0.55; 3.57]	0.481	0.28	[0.06; 1.29]	0.103
High frequency and negative thoughts	7.58	[3.22; 17.83]	**< 0.001**	7.60	[3.19; 18.09]	**< 0.001**
High frequency and positive thoughts	3.39	[0.99; 11.56]	0.051	6.71	[2.13; 21.15]	**0.001**

*CI* confidence interval, *OR* odds ratios

^a^adjusted for sociodemographic (age, sex, education, employment, marital status) and health-related variables (diabetes duration ≥10 years, oral glucose-lowering drugs, insulin, number of comorbidities > 3, perceived health status (fair to poor vs. good to excellent))

## Discussion

### Main findings

The DRM-based questions on thoughts about health proposed by Dolan (2011) were used in the sample of people with DM to measure the frequency of thoughts and to capture the related affective experiences of respondents. Presence of thoughts about health was identified in a number of people with DM in everyday life and was associated with the physical and mental component summary score of the SF-12, diabetes-related distress and depressive symptoms. Surprisingly, people with positive thoughts and negative thoughts alike have a lower HRQoL than people who had not thought about their health the day before. In addition, people with high frequency and positive thoughts have a higher chance of experiencing depressive symptoms than people who had not thought about their health the day before.

### Comparison with other studies

Contrary to Dolan’s (2011) survey of the general population in the USA [21], our study found more people who had not thought about their health the day before (26% vs. 46%). Suppression of unacceptable and/or unwanted repetitive thoughts and impulses defined as intrusive thoughts [45] is reported as a coping strategy for numerous diseases and may be one reason for these differences [46, 47]. On the other hand, a lower percentage of people in the ‘low frequency and positive thoughts’ group (21% vs. 40%) was found. The proportions of people who reported ‘low frequency and negative thoughts’, ‘high frequency and negative thoughts’ and the ‘high frequency and positive thoughts’ were almost identical in both studies.

Contrary to our expectations, the number of people who had thought about their health the day before and had predominantly negative thoughts was similar in both studies, although the target groups were different (general population vs. people with a chronic disease). It is assumed that people with diabetes are more vulnerable to the occurrence of negative thoughts. For example, negative thinking increases if individual consider themselves to be responsible for the disease [42]. Schabert et al. (2013) described how people with type 2 diabetes are confronted with the stigmatisation that they are responsible for their own situation, for example for being overweight or leading an unhealthy lifestyle [48]. Further studies reported concerns about hypoglycaemia and complications in people with DM [49, 50].

We found an association between positive thoughts and a lower HRQoL, which is not consistent with the results of Dolan (2011) where more frequent and positive thoughts were associated with higher valuations of state of health and only more frequent and negative thoughts were associated with lower health state valuations [21, 51]. A possible explanation for our finding may be a negative effect of unrealistically positive thoughts implying excessive expectations, as suggested by MacLeod and Moore [52]. However, comparison is difficult due to different outcome measures (preference-based valuation of HRQoL using Time-Trade-Off method vs. HRQoL measured by the SF-12), the heterogeneity of the study designs (e.g. online and telephone interviews vs. questionnaire) and the different target groups in the studies (general population vs. people with a chronic disease).

In line with other research [2, 7] we found an association between negative thoughts and higher diabetes-related distress and depressive symptoms. One reason for the unexpected associations identified in our study between frequent and positive thoughts and depressive symptoms could be an increased confrontation of diabetes and one’s own health in the case of an existing diagnosis. For instance, there is no increased risk of developing depression in people with prediabetes or undiagnosed diabetes [53]. Nevertheless, the confidence intervals are wide-ranging, which has to be taken into account when interpreting this association.

### Implications for research and practice

This explorative study applied the DRM-based questions on thoughts about health for the first time in the population with DM in order to obtain data on the frequency of thoughts about health and to distinguish between positive and negative thoughts based on an assessment of intensity of feelings. The DRM-based approach focuses on respondents’ actual experiences and represents an alternative to retrospective self-report questionnaires asking respondents about the typical content and/or style of their thinking. It has the potential to overcome the limitations of self-report questionnaires with regard to format (lengths of items and scales) and content validity (constructed experience biased by beliefs, subjective interpretations etc.). However, more research is needed with regard to the validity of the proxy questions collecting DRM-type data.

It was not possible to identify a positive association between thoughts about health and health outcomes. Given the limitations of cross-sectional data, longitudinal studies including various health outcomes (e.g. health promoting behaviours) are needed to better understand long-term consequences of positive and negative thoughts about health.

Given that the present study finds thoughts about health to be associated with worse patient-reported outcomes, a discussion of training in how to deal with thoughts in diabetes education programmes would seem pertinent. Two randomised controlled trials showed that people with type 2 diabetes who were additionally trained to experience their thoughts and feelings instead of changing or stopping them used more coping strategies, reported better diabetes self-management, and had better HbA_1c_ values [5, 54]. Further research must be carried out to discuss the implications of such interventions which may themselves already lead to an increased frequency of thoughts.

### Limitations and strengths

The main limitation of our study is the unproven validity and reliability of the questions used to assess the frequency and valence of thoughts about health. However, the study was designed as an explorative study to test the DRM-based approach to assessing thoughts about health in the population with DM - as proposed by Dolan [21]. The questions therefore still allowed us to collect data on actual experiences (thoughts about health and related feelings) with a very short recall period and without subjective interpretations of response categories by respondents. The ‘U-index method’ [21, 23] which was used to determine the valence of thoughts also helped to overcome respondents’ tendency to avoid extremes such as the answer category ‘very satisfied’. The U-index seems to be a useful method of identifying negative thoughts. However, it has to be mentioned that 16 out of 25 people with high frequency and positive thoughts in our study rated negative and positive thoughts equally. This might have led to a misclassification bias in our results.

Where values were missing in individual items of the questions (*n* = 12), assignment was performed as described in the online resource (Additional file 1, Appendix 1). This may lead to an inaccuracy in a small number of values. Exclusion of participants because of missing values may have caused biases in model fitting and estimation. Furthermore, the multiple regression models were fitted and analysed using the same data, such that associations might be overestimated.

The strengths of this study include the high response rate (51%) of the DiaDec-study, and the large sample size. However, the group of people who reported high frequency and positive thoughts about health (*n* = 25) is still small. The corresponding regression parameter estimates were imprecise with large confidence intervals. However, there was enough power to conclude a significant association with a lower health-related quality of life and depressive symptoms.

A further strength is the possibility of combining primary and secondary data, providing both patient-reported outcomes and valid clinical data.

## Conclusion

The present study shows that thoughts about health, as measured by a variant of the DRM, are a part of everyday life for a substantial number of people with DM. Thoughts about health are associated with a reduced physical and mental component summary score of the SF-12, diabetes-related distress and depressive symptoms. Surprisingly, people with positive thoughts have a reduced HRQoL and people with high frequency and positive thoughts are more likely to experience depression symptoms than people who had not thought about their health the day before. Further research is needed to validate the DRM-based questions measuring thoughts about health in everyday life. That would allow for thoughts about health to then be considered in diabetes education and patient counselling, for instance by teaching patients how to deal with such thoughts. It is also important that particular attention is paid to outcomes of interventions that may themselves lead to an increase in the frequency of thoughts about health.

## Supplementary Information

**Additional file 1: Appendix 1.** Description of data: Visualisation of Dolan’s (2011) ‘thoughts about health’ instrument in the DiaDec-study.

## Data Availability

All available data can be obtained from the corresponding author.

## Abbreviations

DM: Diabetes mellitus
DRM: Day Reconstruction Method
EMA: Ecological Momentary Assessment
HRQoL: Health-related quality of life
PAID: Problem Areas in Diabetes (PAID) instrument
PHQ-9: Patient Health Questionnaire-9
RNT: Repetitive negative thinking
SF-12: 12-Item Short-Form Health Survey
