# Supplementary material for: Thoughts about health and patient-reported outcomes among people with diabetes mellitus: results from the DiaDec-study
Source: BMC Public Health. 2021 Jan 26;21:213. doi: 10.1186/s12889-021-10231-y (PMC7836192; doi:10.1186/s12889-021-10231-y)
Supplement: Supplementary file 1 — Additional file 1: Appendix 1. Description of data: Visualisation of Dolan’s (2011) ‘thoughts about health’ instrument in the DiaDec-study. [file 12889_2021_10231_MOESM1_ESM.docx]

Appendix 1

**Visualisation of Dolan’s ‘thoughts about health’ instrument in the DiaDec-study**

**How often did you think about your health yesterday? ^a^**

|__| not at all

|__| a few times

|__| many times

|__| continually

**If you did think about your health, how did you feel about it? ^b, c, d, e^**

**Please rate each feeling on the scale given.**

**0: does not apply at all; 6: applies exactly**

0 1 2 3 4 5 6

happy |__| |__| |__| |__| |__| |__| |__|

angry |__| |__| |__| |__| |__| |__| |__|

depressed |__| |__| |__| |__| |__| |__| |__|

worried |__| |__| |__| |__| |__| |__| |__|

^a^ The first group included all participants who had not thought about their health the day before. If respondents thought about health ‘a few times’ they were placed in the low frequency group and if they thought about health ‘many times’ or ‘continually’ they were placed in the high frequency group.

^b^ If no rating of feelings was missing and ‘happy’ was the highest or joint-highest rated feeling, thoughts were labelled as ‘positive’, otherwise thoughts were labelled as ‘negative’.

^c^ If the rating of ‘happy’ and of at least one other feeling one higher-rated negative item did not show missing values, the question was coded despite further missing items as ‘negative’. If in this case participants answered ‘happy’ with ‘applies exactly’, the question was coded as ‘positive’ regardless of other missing values.

^d^ If only the rating of ‘happy’ did not show a missing value and participants answered ‘happy’ with ‘applies exactly’, the question was coded as ‘positive’ regardless of other missing values.

^e^ If the rating of ‘happy’ was missing value, then the question was coded as missing.
